# Supplementary material for: Transcriptomic Insights into Mechanisms of Early Seed Maturation in the Garden Pea (Pisum sativum L.)
Source: Cells. 2020 Mar 23;9(3):779. doi: 10.3390/cells9030779 (PMC7140803; doi:10.3390/cells9030779)
Supplement: Supplementary file 1 [file cells-09-00779-s001.zip › Supplementary materials/FileS3.zip!/FileS2/Hormones - Results/Supporting Maps&Profiles/Entropy Profiles.pdf]

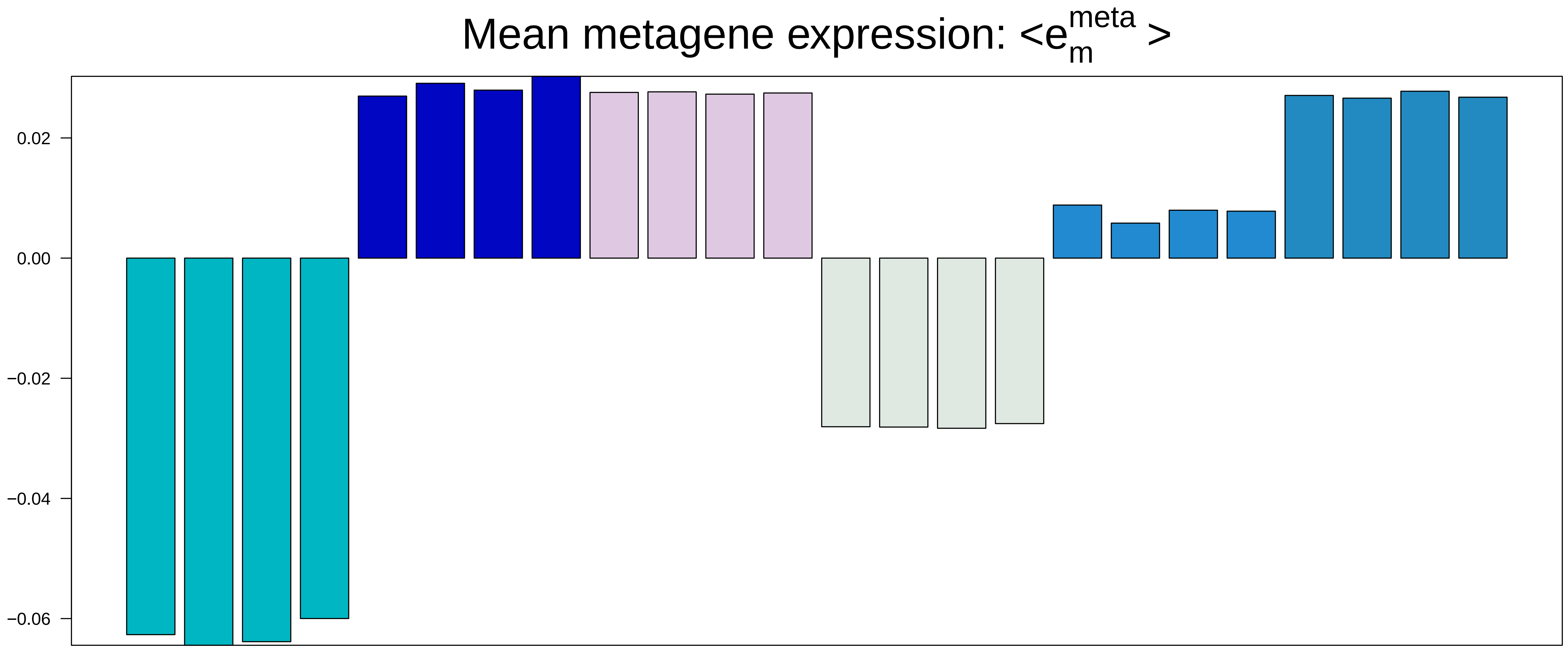
Mean metagene expression: <e$_m^{meta}$ >
0.02
0.00
−0.02
−0.04
−0.06

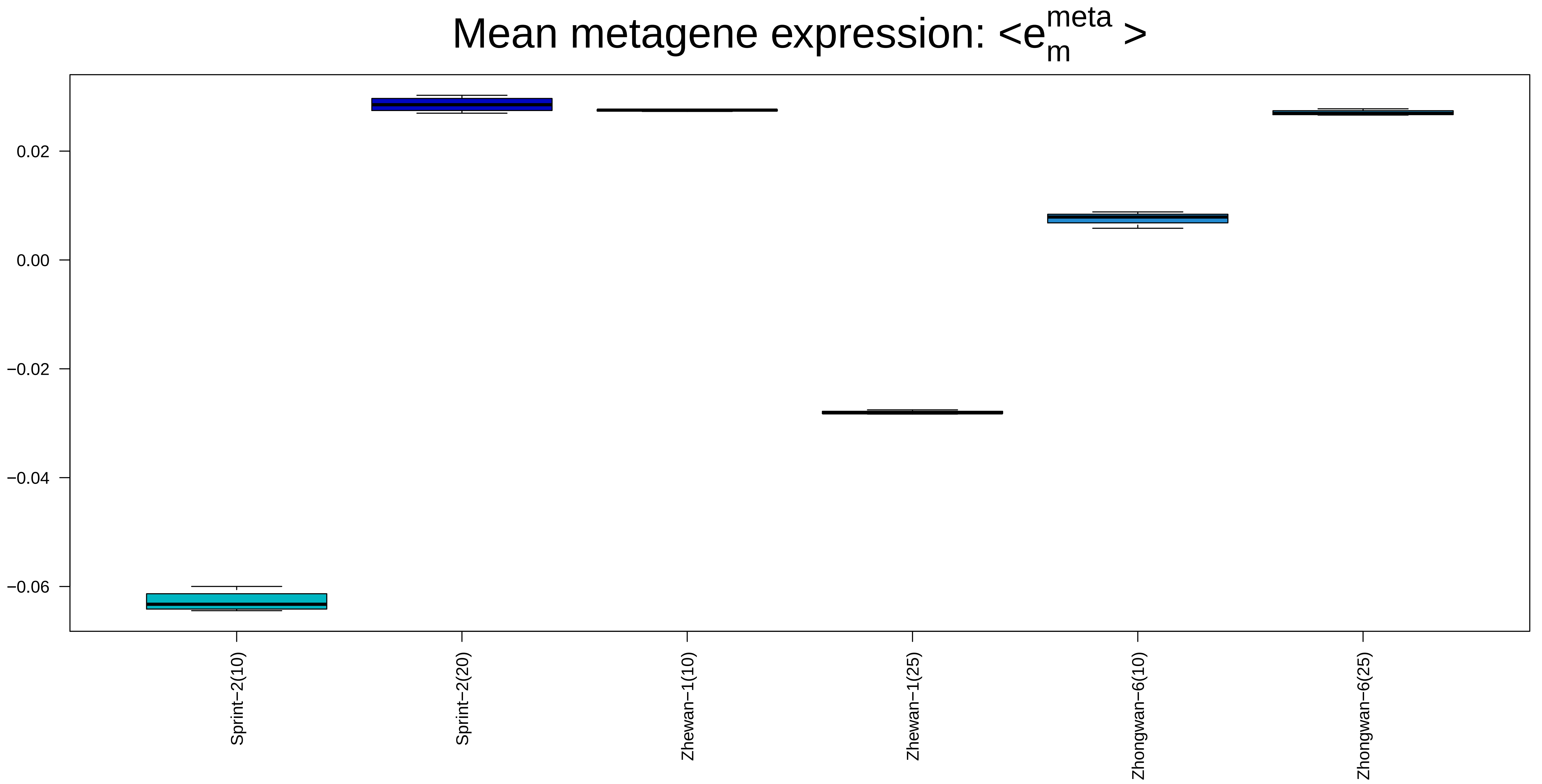
Mean metagene expression: $<e_m^{meta}>$
0.02
0.00
−0.02
−0.04
−0.06
Sprint−2(10)
Sprint−2(20)
Zhewan−1(10)
Zhewan−1(25)
Zhongwan−6(10)
Zhongwan−6(25)

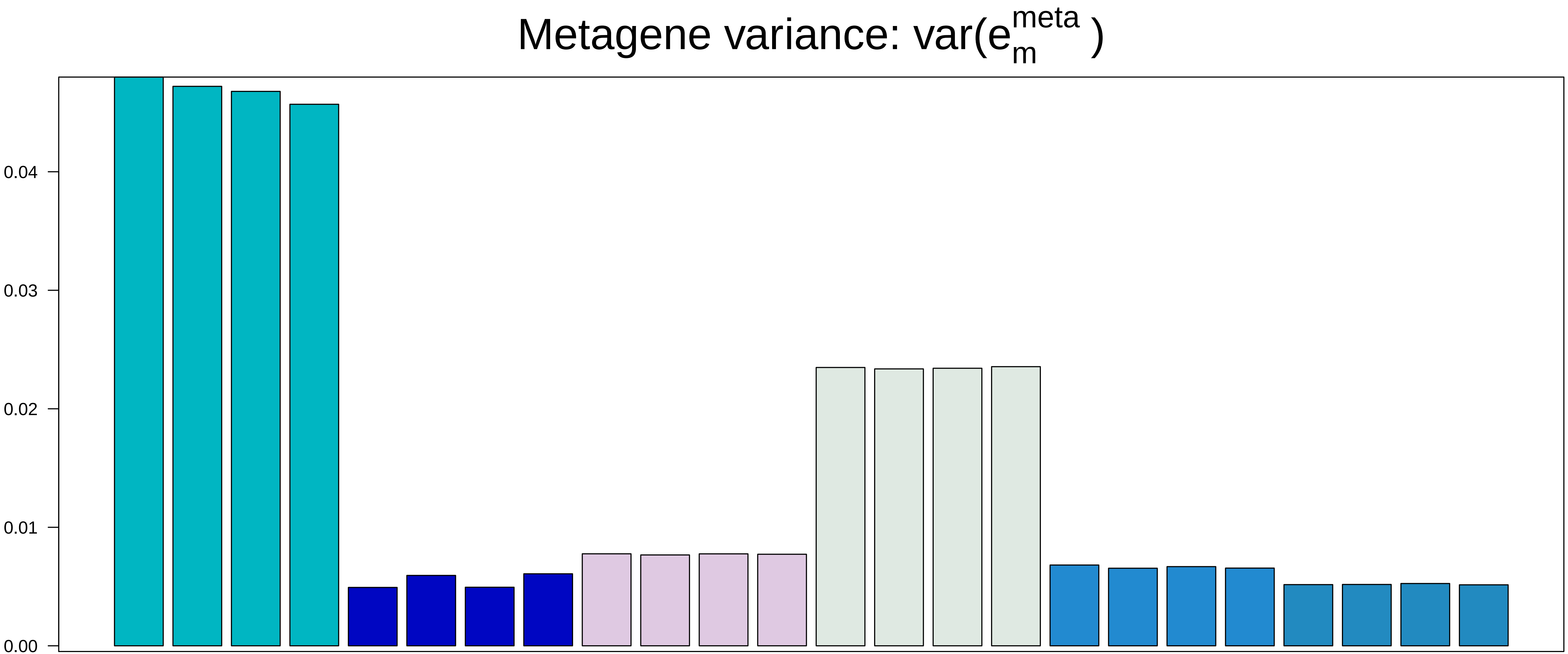
Metagene variance: var(e meta m)
0.04
0.03
0.02
0.01
0.00

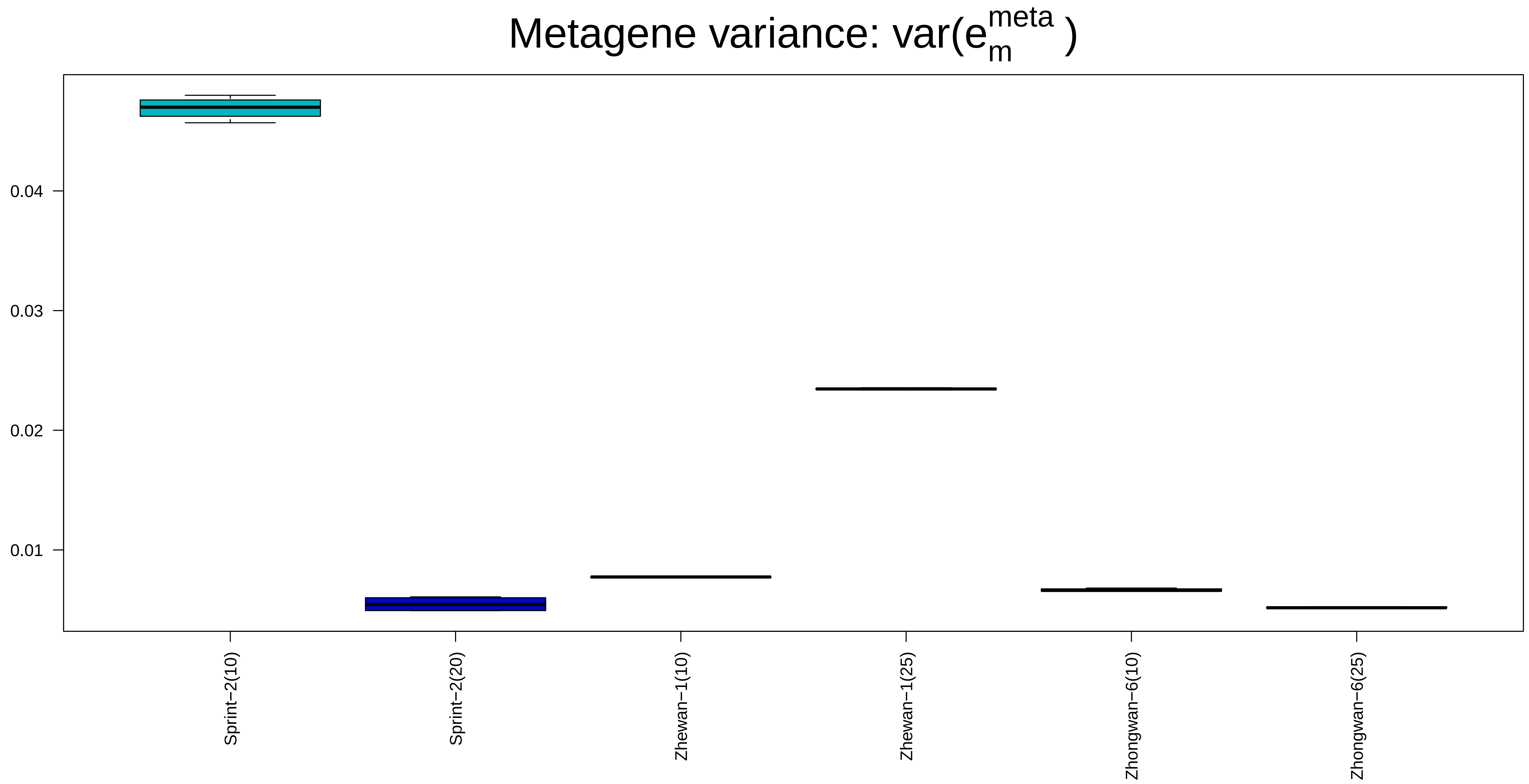
Metagene variance: var(e_m^{meta})
0.04
0.03
0.02
0.01
Sprint−2(10)
Sprint−2(20)
Zhewan−1(10)
Zhewan−1(25)
Zhongwan−6(10)
Zhongwan−6(25)

Standard metagene entropy: h_m

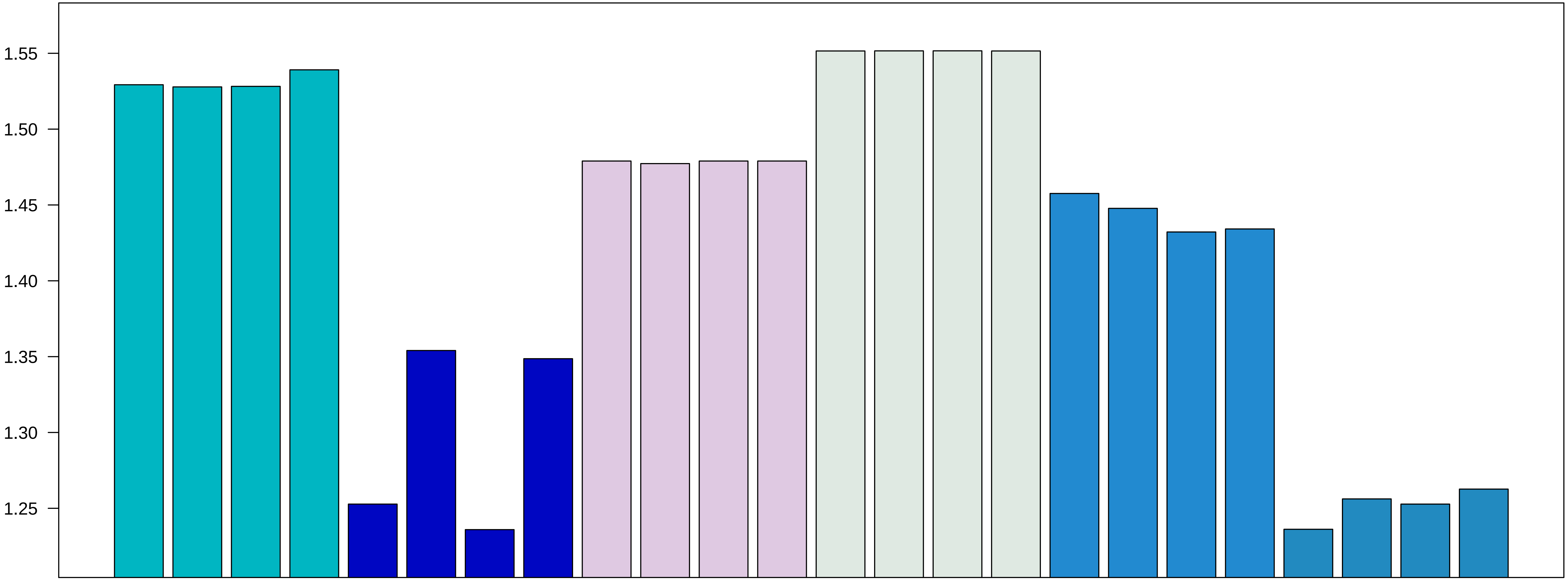

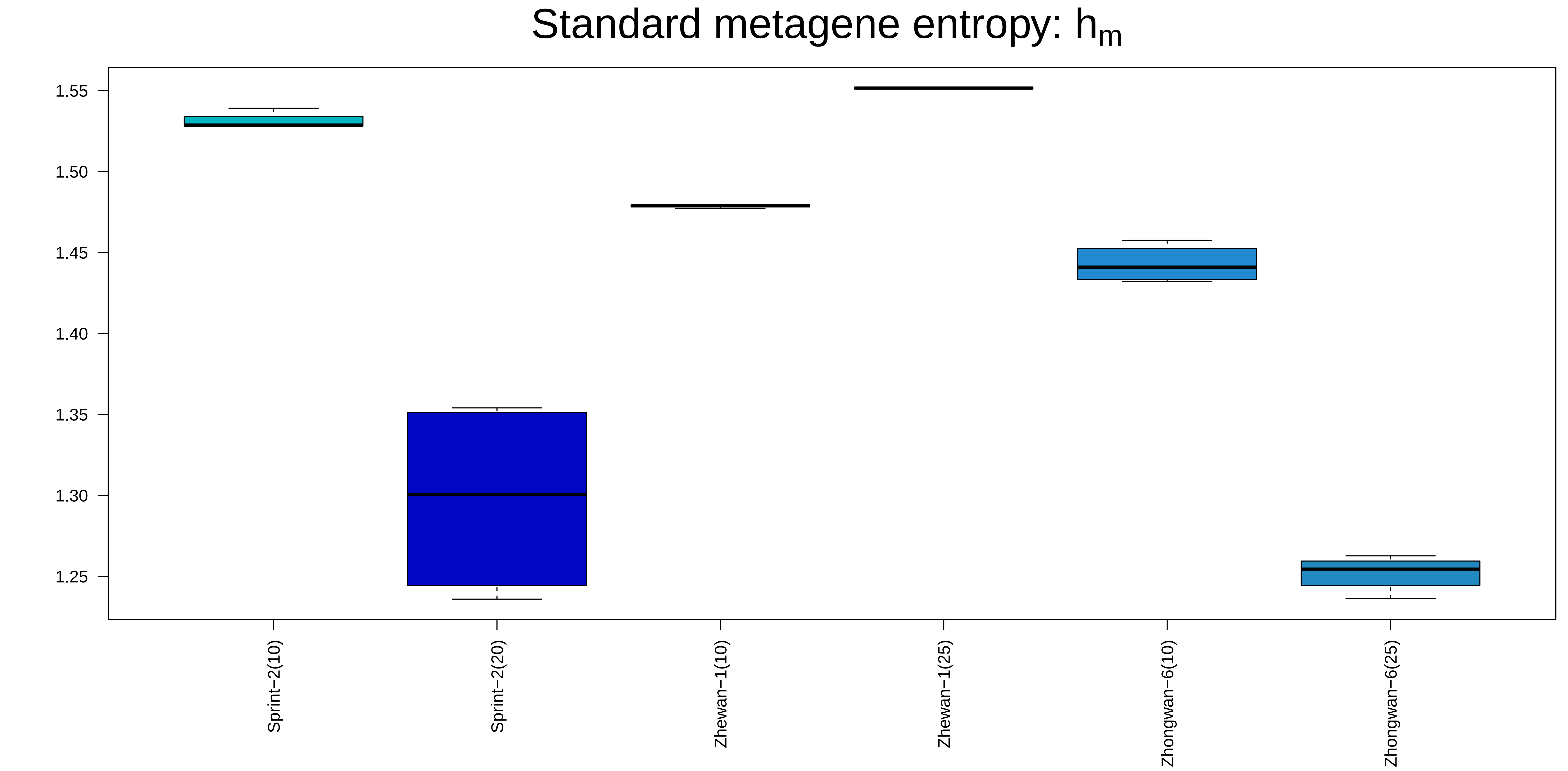
Standard metagene entropy: h_m
1.55
1.50
1.45
1.40
1.35
1.30
1.25
Sprint−2(10)
Sprint−2(20)
Zhewan−1(10)
Zhewan−1(25)
Zhongwan−6(10)
Zhongwan−6(25)

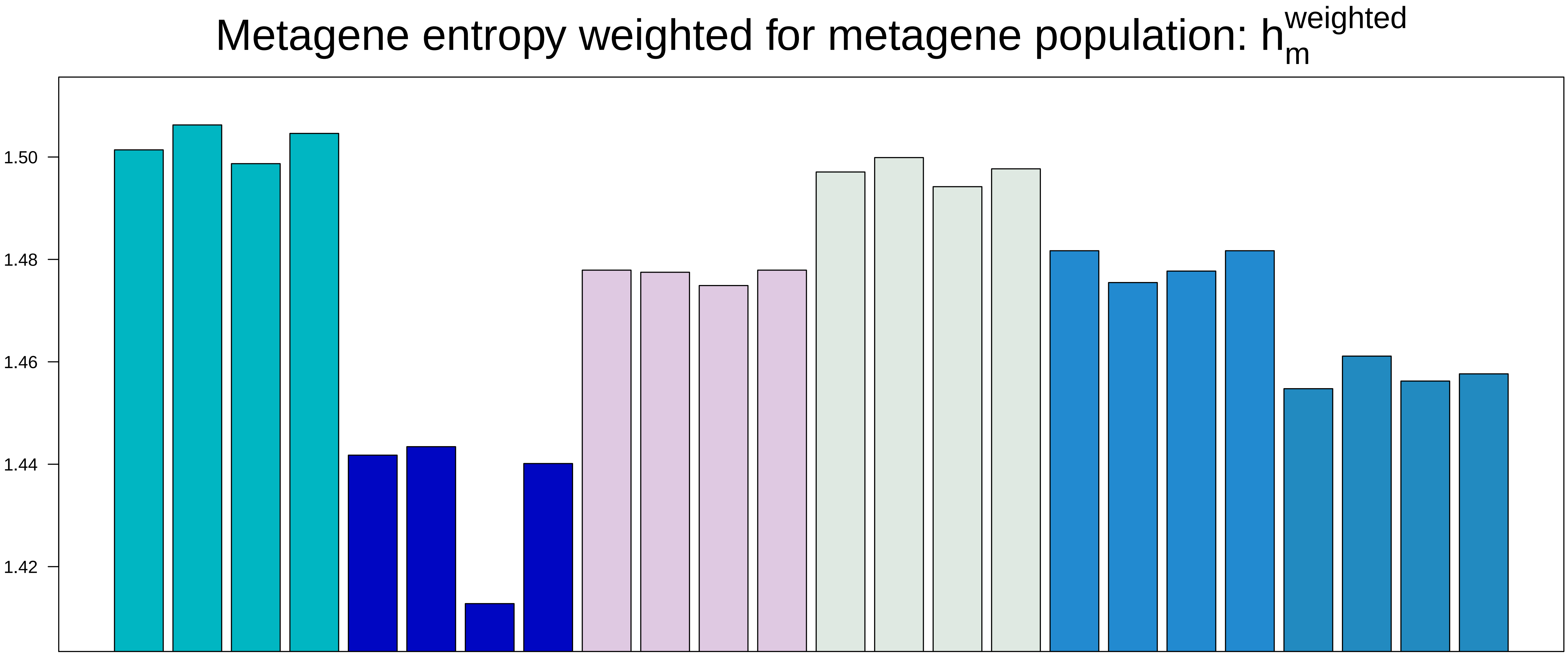
Metagene entropy weighted for metagene population: $h_m^{weighted}$
1.50
1.48
1.46
1.44
1.42

Metagene entropy weighted for metagene population: $h_m^{weighted}$

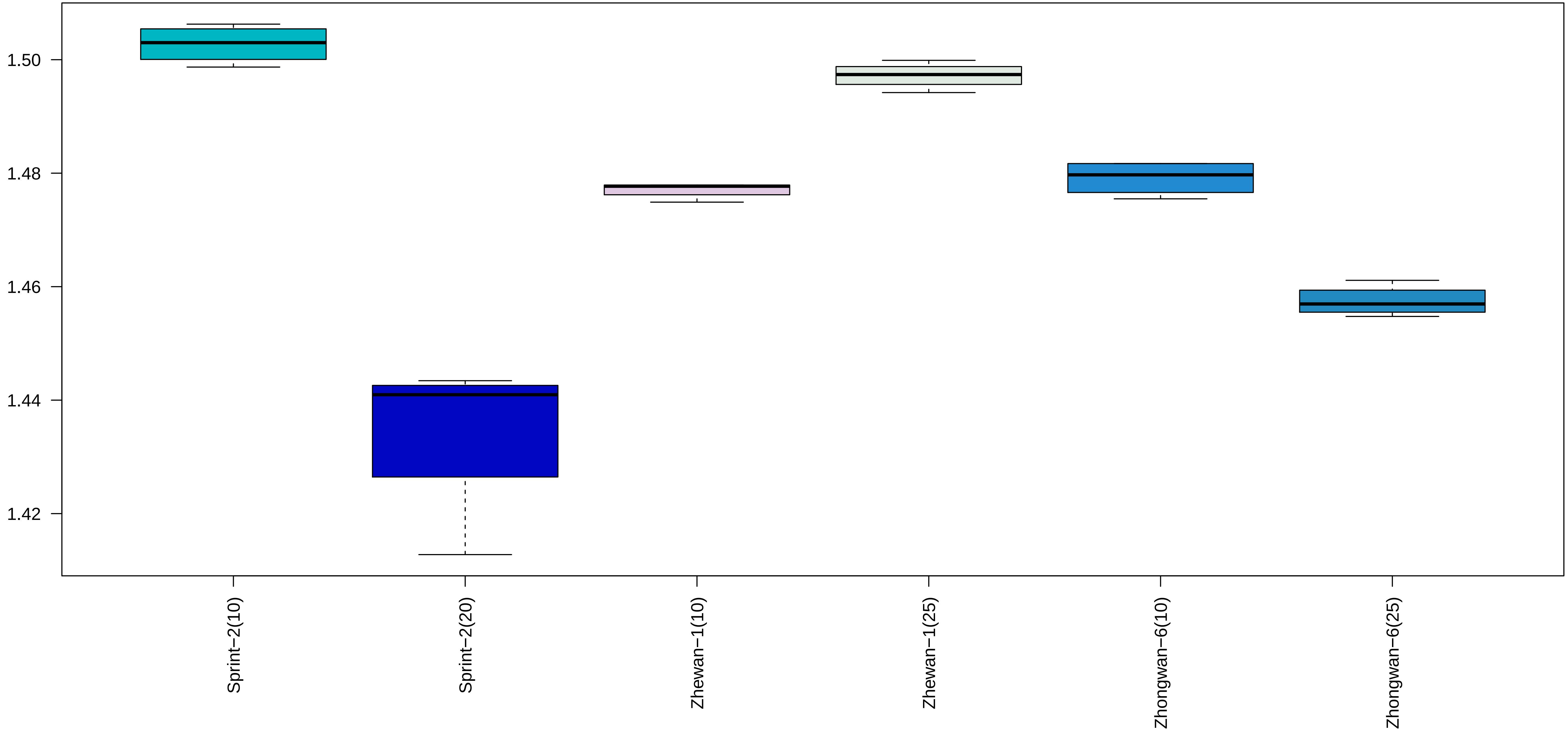

Tsallis metagene entropy: $h_m^{Tsallis}$

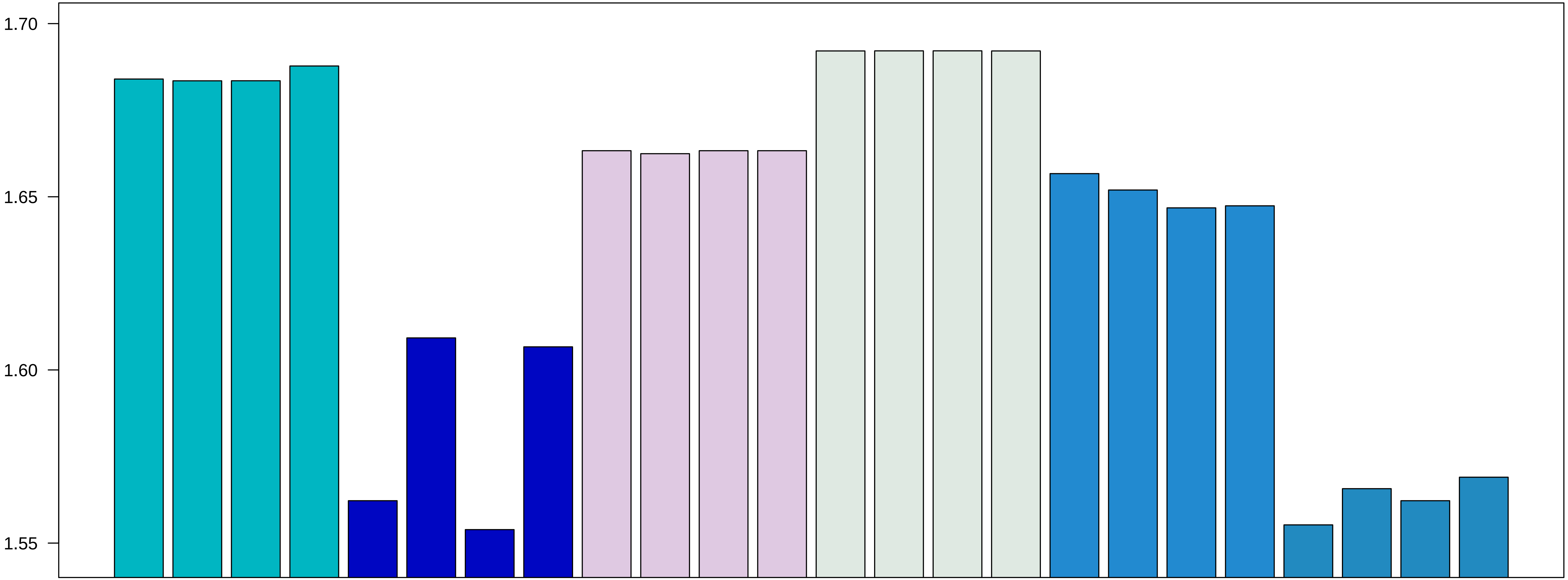

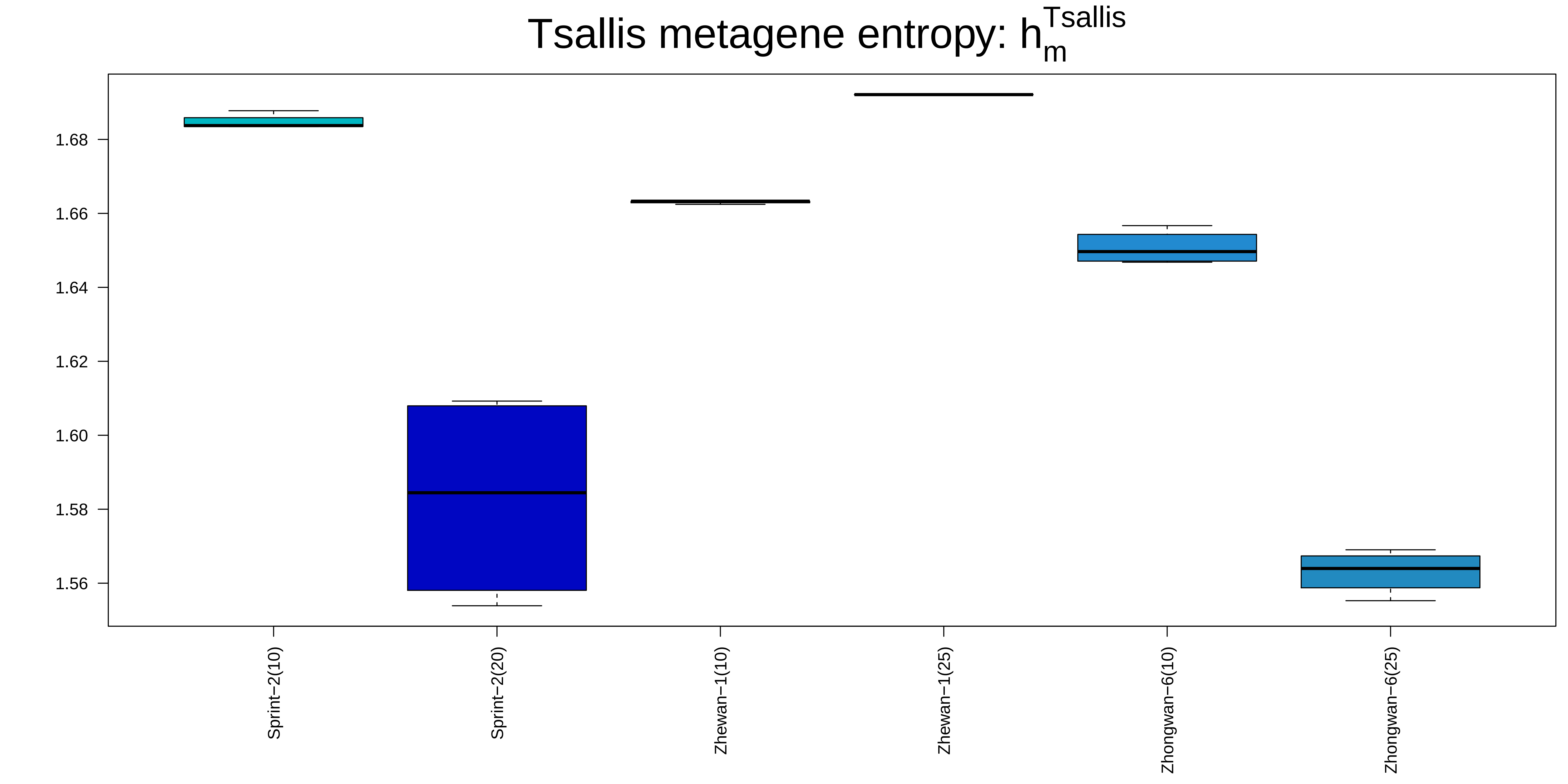

Tsallis metagene entropy: $h_m^{Tsallis}$
1.68
1.66
1.64
1.62
1.60
1.58
1.56
Sprint−2(10)
Sprint−2(20)
Zhewan−1(10)
Zhewan−1(25)
Zhongwan−6(10)
Zhongwan−6(25)
